# Metástase Intracardíaca de Adenocarcinoma Colônico Diagnosticada 12 Anos após Controle do Tumor Primário e sem Sinais de Outras Metástases: Um Relato de Caso

**DOI:** 10.36660/abc.20211014

**Published:** 2022-11-23

**Authors:** Isabela Galizzi Faé, Gabriela Zamunaro Lopes Ruiz, Gustavo Palmer Irffi, Robson de Souza Almeida, Pedro Anjos Conceição, Eduardo Belisario Falchetto, Luiz Guilherme Passaglia, Geraldo Brasileiro, Cláudio Leo Gelape, Clara Rodrigues Alves de Oliveira

**Affiliations:** 1 Hospital das Clínicas da Universidade Federal de Minas Gerais Serviço de Cardiologia e Cirurgia Cardiovascular Belo Horizonte MG Brasil Serviço de Cardiologia e Cirurgia Cardiovascular, Hospital das Clínicas da Universidade Federal de Minas Gerais, Belo Horizonte, MG – Brasil; 2 Hospital das Clínicas da Universidade Federal de Minas Gerais Serviço de Medicina Interna Belo Horizonte MG Brasil Serviço de Medicina Interna, Hospital das Clínicas da Universidade Federal de Minas Gerais, Belo Horizonte, MG – Brasil; 3 Hospital das Clínicas da Universidade Federal de Minas Gerais Serviço de Patologia Belo Horizonte MG Brasil Serviço de Patologia, Hospital das Clínicas da Universidade Federal de Minas Gerais, Belo Horizonte, MG – Brasil; 4 Hospital Felício Rocho Serviço de Cardiologia Belo Horizonte MG Brasil Serviço de Cardiologia, Hospital Felício Rocho, Belo Horizonte, MG – Brasil; 5 Faculdade de Medicina da Universidade Federal de Minas Gerais Departamento de Cirurgia Belo Horizonte MG Brasil Departamento de Cirurgia, Faculdade de Medicina da Universidade Federal de Minas Gerais, Belo Horizonte, MG – Brasil

**Keywords:** Adenocarcinoma, Metástase Neoplásica, Genes Supressores de Tumor, Neoplasias Cardíacas, Neoplasias do Colo, Antígeno Carcinoembrionário, Trombopenia, Diagnóstico por Imagem/métodos

## Introdução

Trombo, vegetação e tumor são os principais diagnósticos diferenciais de massas intracardíacas.^1^ Tumores malignos são raros, enquanto as metástases cardíacas são 20 vezes mais frequentes.^2^ Metástases cardíacas são causadas por disseminação linfática, sanguínea,^3^ invasão direta do mediastino ou crescimento tumoral na veia cava inferior ou nas veias pulmonares^4–6^, levando à obstrução das vias de entrada ou saída de câmaras esquerda ou direita.^7^ Frequentemente, os sintomas são semelhantes aos de outras doenças cardiovasculares, tais como, dispneia, dor torácica, palpitações e edema.^8^ No entanto, por vezes uma massa cardíaca é encontrada incidentalmente durante um exame por imagem realizado por outra indicação.^1^ O objetivo do estudo é descrever um caso incomum de metástase intracardíaca de adenocarcinoma colônico diagnosticada 12 anos após o final do tratamento do tumor primário.

## Apresentação do caso

Um homem de 61 anos de idade foi admitido, em janeiro de 2020, no setor de emergência de um hospital terciário por quadro súbito de dispneia, sudorese, palidez e tontura. Ao exame físico, o paciente apresentava edema de membros inferiores, pressão venosa jugular elevada e sopro sistólico na via de saída do ventrículo direito. Sua história pregressa incluía hipertensão arterial sistêmica, diabetes *mellitus*, doença renal crônica e adenocarcinoma colônico tratado com ressecção cirúrgica e quimioterapia em 2005. Em 2007, uma metástase hepática também foi adequadamente ressectada e, em 2009, o paciente recebeu a última dose de quimioterapia. Foi realizado acompanhamento regular com colonoscopia, tomografias e dosagem do antígeno carcinoembrionário (CEA), sem qualquer sinal de recorrência.

**Figura 1:**
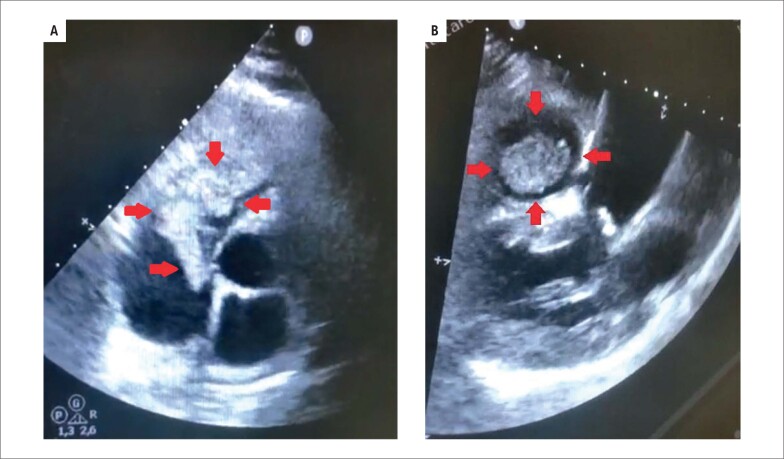
Ecocardiografia. Massa intracavitária (setas) identificada por ecocardiograma transtorácico. Janela quatro câmaras (A) Janela paraesternal eixo longo (B).

A avaliação inicial excluiu infarto agudo do miocárdio e revelou anemia hipocrômica microcítica, deterioração da função renal e trombocitopenia grave (16 x 103/μL). O ecocardiograma transtorácico evidenciou uma enorme massa no ventrículo direito com extensão para o átrio direito e tronco da artéria pulmonar (figuras 1A e B). A tomografia computadorizada (TC) não mostrou evidências de massas pulmonares ou abdominais. Houve apenas uma pequena elevação do CEA (anterior: 2,9 ng/mL em 2019, atual: 6,5 ng/mL). O mielograma revelou medula hiperproliferativa, tendo a hipótese de trombocitopenia atribuída à destruição periférica de plaquetas pela própria massa ou por mecanismo imunológico.

**Figura 2:**
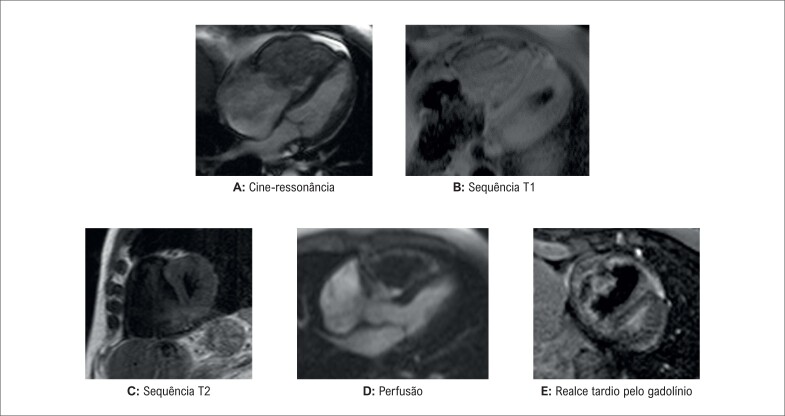
Ressonância magnética cardíaca. Massa intracavitária em diferentes sequências.

A ressonância magnética cardíaca (RMC) evidenciou uma massa intracavitária no ventrículo direito, ocupando a maior parte da cavidade. A massa não apresentava contratilidade intrínseca e estava aparentemente fixada à parede ventricular, sem invasão local. A via de saída do ventrículo direito encontrava-se quase completamente ocupada pelo tumor; havia ainda restrição à abertura da válvula tricúspide e movimento sistólico anormal do septo interventricular para a esquerda. As dimensões da massa eram 8,4 cm (craniocaudal), 4,4 cm (anteroposterior) e 5,7 cm (longitudinal). A caracterização do tecido demonstrou uma aparência heterogênea em todas as sequências. Na cine-ressonância, a massa apresentou hipossinal em comparação com o miocárdio, isossinal em T1 e hipossinal em T2, sem mudanças de sinal em sequências com supressão de gordura. Em sequências de perfusão, foi possível observar heterogeneidade e discreto realce por contraste. No realce tardio pelo gadolínio, um hipersinal periférico com um orifício de hipossinal sugeriu que a massa poderia ter um núcleo necrótico ou um componente trombótico (figuras 2 A–E). Devido à trombocitopenia grave, o tratamento com anticoagulação não foi iniciado.

**Figura 3:**
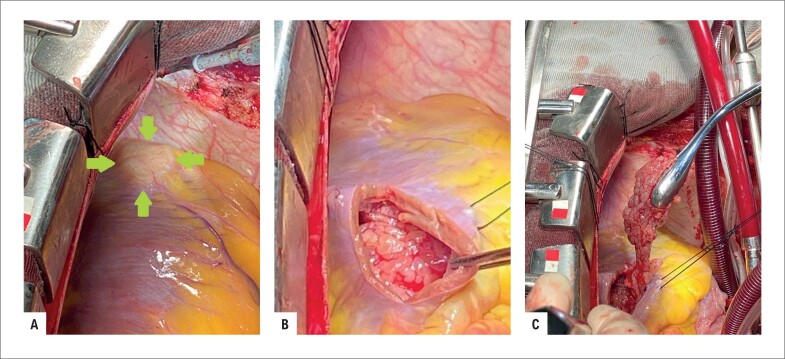
Vista peroperatória. A. Invasão tumoral na parede do ventrículo direito. B. Massa polipoide de tecido mole na cavidade do ventrículo direito. C. Tumor sendo retirado do ventrículo.

Com o objetivo de confirmar a natureza da massa e desobstruir o fluxo sanguíneo, foi programada uma ressecção cirúrgica. Durante a cirurgia, foram identificados sinais de envolvimento miocárdico extenso, além de invasão da parede do ventrículo direito. Foi retirada uma grande quantidade de tumor friável pelo átrio direito (figuras 3 A–C). Devido à impossibilidade de remover completamente a massa pelo átrio direito, foi realizada uma ventriculotomia direita na tentativa de ressecar completamente a lesão, com sucesso parcial devido à invasão da parede. Ecocardiograma transesofágico realizado durante a cirurgia mostrou melhora hemodinâmica após a retirada parcial do tumor.

**Figura 4:**
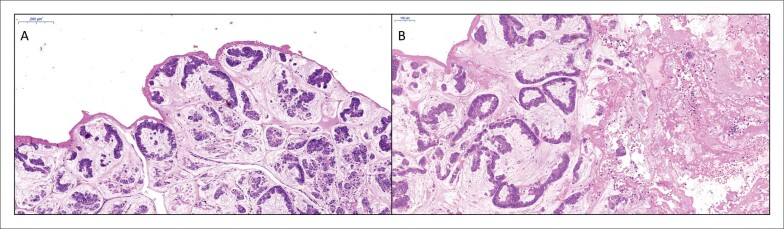
Aspectos microscópicos da lesão. A. Várias glândulas malignas associadas a substância mucosa abundante. Na superfície, há uma fina camada de fibrina. B. Do lado esquerdo da figura, há glândulas malignas entremeadas à substância mucosa; à direita, há material necrótico extenso misturado com fibrina e hemácias.

A amostra cirúrgica consistiu em vários fragmentos irregulares, necróticos e moles. Histologicamente, a lesão foi caracterizada como neoplasia maligna do tipo adenocarcinoma com aspecto mucossecretor/mucinoso,9 com áreas extensas de necrose e autólise, no interior e na superfície dos fragmentos (Figura 4 A–B). Não foi possível avaliar a infiltração miocárdica, já que não foram identificados cardiomiócitos nos vários fragmentos examinados. O estudo imuno-histoquímico demonstrou: CK7, CD20 e KRAS negativos; CDX2 e β-catenina positivos; alto índice de proliferação celular (Ki-67=> 50%). O diagnóstico final foi de metástase cardíaca de adenocarcnimoma colônico. O aspecto microscópico da lesão era semelhante ao do tumor colônico primário ressecado em 2005 e ao da metástase hepática retirada em 2007.

O paciente recebeu alta algumas semanas após a cirurgia, sem sintomas cardiovasculares, níveis de plaqueta normais e com boa recuperação renal. Foi prescrito um ciclo de quimioterapia paliativa, mas o tratamento foi interrompido pela queda do nível de plaquetas. Após um mês, o paciente foi readmitido no hospital com sinais de insuficiência cardíaca descompensada. Foi identificado derrame pleural importante e o ecocardiograma mostrou recidiva da massa, a qual ocupava grande parte do ventrículo direito. Dessa forma, o paciente, sua família e a equipe médica optaram por cuidados paliativo. O paciente faleceu poucas semanas depois por complicações da neoplasia.

O material suplementar mostra a linha do tempo dos eventos desde a admissão do paciente até sua morte.

## Discussão

Tumores cardíacos malignos são raros, especialmente os primários. Metástases cardíacas se originam principalmente de carcinomas de pulmão, mama e esôfago, melanomas, linfomas e leucemias.^3^ Dada a sua baixa incidência, tumores cardíacos não são comumente investigados na prática oncológica, embora tenham ganhado importância graças à melhoria diagnóstica de neoplasias e maior sobrevida dos pacientes.^10^

Tumores cardíacos geralmente evoluem silenciosamente por anos, sendo subdiagnosticados.^11^ Quando sintomáticos, podem causar manifestações constitucionais, obstrução ao fluxo sanguíneo, disfunção valvar, arritmias, derrame pericárdico e embolização.^12^ Implantes cardíacos secundários são frequentemente associados à fase terminal de neoplasia maligna generalizada ou, menos comumente, são descobertas como manifestação inicial de um câncer recentemente diagnósticado.^13^ Quando a neoplasia forma uma massa intracardíaca de dimensões consideráveis, os pacientes apresentam instabilidade hemodinâmica e têm prognóstico pior antes, durante e após uma intervenção cirúrgica.^5^ Infiltração da parede cardíaca por células malignas pode comprometer a dinâmica cardíaca e resultar em evento sintomático e catastrófico para o paciente.^14^

Embora se possa suspeitar de metástases cardíacas em vida, raramente elas são diagnosticadas antes do óbito.^15^ Em estudos de autópsias, metástases endocárdicas de tumores colorretais são detectadas em 1,4 a 7,2% dos pacientes com essa malignidade.^16^ Na análise de Oneglia et al.,^10^ metástases cardíacas foram encontradas em 3,2% dos pacientes autopsiados com carcinoma colorretal conhecido.^10^

O diagnóstico diferencial pré-operatórios de massas cardíacas nem sempre é fácil ou possível, mesmo com acesso fácil a propedêuticas avançadas.^17^ A ecocardiografia é geralmente a primeira ferramenta a ser usada para o diagnóstico de tumores cardíacos.^1^ TC e RMC são importantes, pois oferecem informações anatômicas e visualizam infiltração ou extensão do tumor.^15^ Em muitos pacientes, contudo, o diagnóstico preciso só é confirmado após o exame anátomopatológico da amostra retirada cirurgicamente. Quando a lesão está no átrio e/ou ventrículo direito, pode-se tentar uma biópsia endomiocárdica (BEM). Entretanto, nem sempre a biópsia permite um diagnóstico definitivo, especialmente devido à dificuldade em se obter porções representativas da lesão. No presente caso, o diagnóstico pré-operatório por BEM somente poderia ser possível se um fragmento como o mostrado na Figura 4A pudesse ser obtido. Necrose tumoral extensa, especialmente nas porções superficiais dos fragmentos, faz com que a amostragem das células neoplásicas por BEM seja mais difícil de se alcançar.

A incidência de malignidade colorretal e de outros tumores tem aumentado nos últimos anos.^9^ Com tratamentos oncológicos mais avançados e sobrevida mais prolongada dos pacientes, espera-se que as metástases cardíacas aumentem. Nesse contexto, a equipe médica terá de enfrentar a decisão de realizar ou não cirurgia cardíaca para diagnóstico ou para alívio hemodinâmico dos pacientes com tumores irressecáveis.

O rastreio de metástases cardíacas atualmente não é recomendado para pacientes com neoplasias malignas. Entretanto, pacientes oncológicos que apresentam sintomas cardiopulmonares devem ser avaliados quanto a implantes cardíacos secundários.^15^ Benefícios possíveis da cirurgia cardíaca devem ser contrabalançados em relação à morbimortalidade perioperatória,^18^ assim, cardiologistas, cirurgiões cardiovasculares, oncologistas e outros profissionais devem estar envolvidos na decisão final a respeito do melhor tratamento em cada cenário específico.^15^

Apesar de todas as estratégias, a sobrevida média para pacientes com câncer colorretal metastático não ressecável que recebem o melhor tratamento de suporte é de apenas cinco a seis meses. Em geral, pacientes com metástases cardíacas têm uma taxa de sobrevida em cinco 5 anos de apenas 7%.^19^ Com a melhoria na detecção precoce, desenvolvimento de ferramentas diagnósticas modernas, avanços nos regimes de quimioterapia, técnicas de radiação e refinamento dos cuidados pré-operatórios, estima-se que haja um aumento na sobrevida dos pacientes oncológicos.^15^

## *Material suplementar

Para informação adicional, por favor, clique aqui.
