# Supplementary material for: Metástase Intracardíaca de Adenocarcinoma Colônico Diagnosticada 12 Anos após Controle do Tumor Primário e sem Sinais de Outras Metástases: Um Relato de Caso
Source: Arq Bras Cardiol. 2022 Nov 23;119(6):991–5. [Article in Portuguese] doi: 10.36660/abc.20211014 (PMC9814808; doi:10.36660/abc.20211014)
Supplement: Translation

# Translation: Intracardiac Metastasis of Colonic Adenocarcinoma 12 Years After Primary Tumor Control and Without Any Sign of Other Metastasis: A Case Report

**Authors:** Isabela Galizzi Faé, Gabriela Zamunaro Lopes Ruiz, Gustavo Palmer Irffi, Robson de Souza Almeida, Pedro Anjos Conceição, Eduardo Belisario Falchetto, Luiz Guilherme Passaglia, Geraldo Brasileiro, Cláudio Leo Gelape, Clara Rodrigues Alves de Oliveira

**Affiliations:** 1 Belo Horizonte MG Brazil Serviço de Cardiologia e Cirurgia Cardiovascular, Hospital das Clínicas da Universidade Federal de Minas Gerais, Belo Horizonte, MG – Brazil; 2 Belo Horizonte MG Brazil Serviço de Medicina Interna, Hospital das Clínicas da Universidade Federal de Minas Gerais, Belo Horizonte, MG – Brazil; 3 Belo Horizonte MG Brazil Serviço de Patologia, Hospital das Clínicas da Universidade Federal de Minas Gerais, Belo Horizonte, MG – Brazil; 4 Belo Horizonte MG Brazil Serviço de Cardiologia, Hospital Felício Rocho, Belo Horizonte, MG – Brazil; 5 Belo Horizonte MG Brazil Departamento de Cirurgia, Faculdade de Medicina da Universidade Federal de Minas Gerais, Belo Horizonte, MG – Brazil

**Keywords:** Adenocarcinoma, Neoplasm Metastasis, Genes, Tumor Suppresor, Heart Neoplasms, Colonic Neoplasms, Carcinoembryonic Antigen, Thrombocytopenia, Diagnostic, Imaging/methods

## Introduction

Thrombus, vegetation and tumor are the main diagnoses for intracardiac masses.^1^ Malignant cardiac tumors are rare and cardiac metastasis are around 20 times more frequent than primary tumors.^2^ Cardiac metastases originate from lymphatic or blood dissemination,^3^ mediastinum direct invasion or tumoral growth inside inferior vena cava or pulmonary veins^4–6^ and can lead to obstruction of the right or left outflow or inflow tract or arrhythmias.^7^ Frequently, symptoms are similar to other prevalent cardiovascular diseases, such as dyspnea, chest pain, palpitations and edema,^8^ but sometimes a cardiac mass is found out incidentally during an image exam performed for an unrelated indication.^1^ We aim to describe an uncommon case of intracardiac metastasis from colonic adenocarcinoma 12 years after the end of the primary tumor treatment.

## Case presentation

A 61-years-old man was admitted, in January 2020, at the emergency department of a Brazilian tertiary hospital due to a sudden crisis of dyspnea, sweating, pallor and dizziness. On physical examination, the patient had symmetric lower limbs edema, elevated jugular venous pressure and systolic heart bruit in the right ventricular outflow tract. His medical history included hypertension, *diabetes mellitus*, chronic kidney disease and colonic adenocarcinoma treated by surgical resection and chemotherapy in 2005. In 2007, a hepatic metastasis was also properly resected and, in 2009, he received the last dose of chemotherapy. He was regularly followed up with colonoscopy and carcinoembryonic antigen assessment, without any sign of recurrence.

The initial evaluation ruled out acute myocardial infarction and showed hypochromic and microcytic anemia, worsening renal function, and severe thrombocytopenia (16 x 10^3^/μL). A transthoracic echocardiogram described a huge mass in the right ventricle with extension to the right atrium and pulmonary trunk (figures 1A and B). Computed Tomography (CT) scan showed no evidence of pulmonary or abdominal masses. There was only a minor elevation of the carcinoembryonic antigen (CEA) (previous: 2.9 ng/mLin 2019, current: 6.5 ng/mL). A myelogram showed a hyperproliferative bone marrow, so the hypothesis of thrombocytopenia was attributed to peripheral platelets destruction by the tumor itself or by immunological mechanism.

**Figure 1:** Echocardiography. Intracavitary mass (arrows) identified by transthoracic echocardiogram in four chambers view (A) and parasternal long axis view (B).

A cardiac magnetic resonance (CMR) showed an intracavitary mass in the right ventricle, occupying most part of its cavity. The mass didn't have intrinsic contractility and was apparently fixed in the ventricular wall, without local invasion. The outflow tract of the right ventricle was almost completely occupied by the tumor, tricuspid valve opening was significantly restricted and there was systolic abnormal leftward motion of interventricular septum. Dimensions of the mass were 8.4 cm (craniocaudal), 4.4 cm (anteroposterior) and 5.7 cm (longitudinal). Tissue characterization showed heterogeneous appearance in all sequences. At cineresonance, the mass exhibited hyposignal in comparison with myocardium, isosignal in T1 and hyposignal in T2, without signal changes in sequences with fat suppression. In perfusion sequences, heterogeneity and discrete contrast enhancement could be seen. In late gadolinium enhancement, a peripheric hypersignal with a hyposignal hole suggested the mass could have a necrotic core or a thrombotic component (figures 2 A–E). Because of the severe thrombocytopenia, anticoagulation therapy was not initiated.

**Figure 2:** Cardiac magnetic resonance. Intracavitary mass in different sequences in CMR.

Aiming to confirm the mass etiology and unblock the blood flow, a surgical resection was scheduled. During the surgery, signs of extensive myocardial involvement were identified. The right ventricle wall was invaded and occupied by a large mass of soft tissue. Through the right atrium, a large amount of friable tumor material was removed (figures 3 A–C). Due to the impossibility of complete removal of the mass through the right atrium, right ventriculotomy was performed attempting to completely resect the lesion, with partial success due to wall invasion. A transesophageal echocardiogram was performed during the surgery and showed an improvement in cardiac dynamics after partial tumor removal.

**Figure 3:** Per operative view. A. Tumoral invasion in the right ventricle wall. B. A soft tissue and polypoid mass is seen in right ventricle cavity. C. Tumor being removed from the ventricle.

The surgical sample consisted of many irregular, soft and extensive necrotic fragments. Histologically, the lesion was represented by an adenocarcinoma-type malignant neoplasm of mucosecretory/mucinous aspect,^9^ with extensive areas of necrosis and autolysis, both inside and on the surface of the fragments (Figure 4 A–B). It was not possible to assess myocardial infiltration, since cardiomyocytes were not identified in the various fragments examined. The immunohistochemical study showed: negative CK7, CD20 and KRAS; CDX2 and β-catenin positive; high rate of cell proliferation (Ki-67 => 50%). The final diagnosis was cardiac metastasis from colonic adenocarcinoma. The microscopic aspect of the lesion was similar to the primary colonic tumor resected in 2005 and to the liver metastasis removed in 2007.

**Figure 4:** Microscopic aspects of the lesion. A. Several malignant glands associated with abundant mucous substance. On the surface, there is a thin fibrin layer. B. On the left side of the figure, there are malignant glands inside abundant mucous substance; on the right side, there is extensive necrotic material mixed with fibrin and blood cells.

The patient was discharged a few weeks after the surgery, with no more cardiovascular symptoms, normal platelet levels and renal recovery. One cycle of palliative chemotherapy was prescribed, but treatment was interrupted because of dropping in platelet counts. One month later he was readmitted to the hospital with signs of decompensated heart failure. Right pleural effusion was identified, and echocardiogram showed a mass occupying a large part of the right ventricle. Therefore, the patient, his family and medical team decided for palliative support. He died a few weeks later.

The supplementary material shows the timeline of events since the admission until the patient's death.

## Discussion

Malignant cardiac tumors are rare, particularly the primary ones. Cardiac metastases originate mainly from lung, breast and esophageal carcinomas, melanomas, lymphomas and leukemias.^3^ Due to their low incidence, cardiac tumors are not commonly investigated in oncology practice, although they have been gaining greater importance due to improvement in cancer diagnosis and longer patients survival.^10^

Clinically, cardiac tumors often evolve silently for years, being underdiagnosed,^11^ or can cause constitutional symptoms, obstruction of intracardiac blood flow, valve dysfunction, arrhythmias, pericardial effusion and embolization.^12^ Secondary cardiac implants are frequently associated with the terminal phase of a widespread malignancy or, less commonly, can be the initial manifestation of a newly diagnosed or newly recurrent malignancy.^13^ When neoplastic infiltration forms a large intracardiac mass, the patients present with hemodynamic instability and have a worse prognosis before, during or after surgical intervention.^5^ Infiltration of the cardiac wall by malignant cells can result in a catastrophic and symptomatic event for the patient, impairing cardiac dynamics.^14^

Although cardiac metastases can be suspected in life, they are rarely diagnosed before death.^15^ In autopsy studies, endocardial metastases from colorectal tumors are detected in 1.4 to 7.2% of the patients with this malignancy.^16^ In the review by Oneglia et al.^10^ heart metastases were found in 3.2% of autopsied patients with known colorectal carcinoma.^10^

Preoperative differential diagnosis of cardiac masses is not always easy or possible, even with advanced propaedeutic facilities.^17^ Echocardiography is usually the first tool used for the diagnosis of cardiac tumors.^1^ CT scans and CMR are important to provide anatomic information and to visualize infiltration or extension of the tumor.^15^ In many patients, the precise diagnosis is only confirmed after anatomopathological examination of the surgically removed sample. When the lesion is in the atrium and/or the right ventricle, endomyocardial biopsy may be attempted. However, it does not always allow definitive diagnosis, especially due to the difficulty in obtaining representative portions of the lesion. In the present case, the preoperative diagnosis by endomyocardial biopsy could be possible only if a fragment like that showed in the Figure 4A could be obtained. The extensive tumor necrosis, especially in the superficial portions of the fragments, make the sampling of cells and neoplastic glands by endomyocardial biopsy less attainable.

The incidence of colorectal and other malignancies has been increasing in recent years.^9^ With better cancer treatments and longer patient's survival, cardiac metastases are expected to increase. In this setting, medical staff will be faced with the decision as to perform or not a surgery, for diagnosis or for palliation and improvement of the patient's hemodynamics.

Screening for cardiac metastases is not currently recommended for patients with malignancies. However, oncologic patients presenting with cardiopulmonary symptoms should be evaluated for secondary cardiac implants.^15^ Possible benefits from cardiac surgery may be counterbalanced by perioperative morbimortality^18^ and patients, cardiologists, cardiovascular surgeons, and oncologists should be involved in making the final decision about the best treatment in each specific scenario.^15^

Despite of all the strategies, the median overall survival for patients with unresectable metastatic colorectal cancer who receive best supportive care alone is approximately five to six months. In general, patients with cardiac metastases have a 5-year survival rate of only 7%.^19^ With the improvement of early detection, development of modern diagnostic tools, advances in chemotherapeutic regimens and radiation techniques and refinement of preoperative care, cancer patient survival is expected to increase.^15^

## *Supplemental Materials

For additional information, please click here.
